# RNLS promotes ovarian cancer growth and inhibits ferroptosis via mediating STAT3

**DOI:** 10.3389/fonc.2025.1604377

**Published:** 2025-07-29

**Authors:** Liang Lin, Zuolian Xie, Peilin Zhong, Jian Chen, Ning Ma, Ling Li, An Lin, Li Chen

**Affiliations:** Department of Gynecology, Clinical Oncology School of Fujian Medical University, Fujian Cancer Hospital, Fuzhou, China

**Keywords:** ovarian cancer, RNLS, STAT3, PI3K/AKT, oxidative stress

## Abstract

**Introduction:**

Ovarian cancer (OC) is a lethal malignancy for which there are limited therapeutic options. The role of renalase (RNLS) in cancer progression and ferroptosis regulation remains unclear. This study investigates how RNLS mediates STAT3 to promote OC growth and suppress ferroptosis.

**Methods:**

RNLS expression was analyzed in OC cell lines (OVCAR3) and normal ovarian epithelial cells (IOSE80) via qPCR. Stable RNLS knockdown (sh-RNLS) and overexpression (ov-RNLS) OVCAR3 models were established via lentiviral infection. STAT3 siRNA was transfected to explore RNLS-STAT3 interactions. Functional assays (CCK8, wound healing, Transwell, flow cytometry) evaluated proliferation, migration, invasion, apoptosis, and ROS levels. Mitochondrial morphology was assessed by electron microscopy. Subcutaneous tumor models in mice validated *in vivo* effects. Molecular markers (STAT3, p-PI3K/PI3K, p-AKT/AKT, Ki-67, MDA, GPX4, GSH) were analyzed via Western blot, immunohistochemistry, and ELISA.

**Results:**

RNLS was significantly upregulated in OC cells, particularly OVCAR3. RNLS knockdown suppressed STAT3 expression, cell proliferation, migration, invasion, and tumor growth, while promoting apoptosis, ROS accumulation, and mitochondrial damage. Conversely, RNLS overexpression exerted opposing effects. STAT3 silencing inhibited PI3K/AKT signaling and ferroptosis resistance, which were rescued by RNLS overexpression. *In vivo*, sh-RNLS reduced tumor volume/weight, as well as RNLS/STAT3, Ki-67, GPX4, and GSH, while increasing MDA. ov-RNLS enhanced tumor growth and reversed these molecular changes.

**Conclusion:**

RNLS drives OC progression by activating STAT3-dependent PI3K/AKT signaling, enhancing proliferation, metastasis, and ferroptosis suppression. Targeting RNLS-STAT3 axis may offer a novel therapeutic strategy against OC.

## 1 Introduction

Ovarian cancer (OC) is the deadliest gynecological malignancy. It presents therapeutic challenges primarily due to its tendency to metastasize to the peritoneum and develop resistance to chemotherapy (1). Although targeted therapies such as PARP inhibitors and anti-angiogenic agents have partially improved clinical outcomes, over 70% of advanced-stage patients remain at risk of recurrence (2, 3). This treatment resistance is fundamentally linked to aberrant activation of survival pathways, metabolic reprogramming, and evasion of programmed cell death (4). Consequently, delving into the molecular mechanisms underpinning OC initiation and progression, particularly identifying key regulatory factors driving malignant progression, metastatic dissemination, and therapeutic resistance, and elucidating their functional networks, holds immense clinical significance for developing novel therapeutic targets and improving patient prognosis.

Renalase (RNLS) is a relatively recently identified flavin adenine dinucleotide (FAD)-dependent amine oxidase. Structurally, the RNLS protein contains a characteristic FAD-binding domain and an amine oxidase domain (5).For instance, Hollander et al. demonstrated that elevated RNLS expression in melanoma specimens is inversely with disease-specific survival (6). Pointer et al. further reported that RNLS upregulation in pancreatic and human carcinoma tissues promotes cancer cell survival, thereby shortening patient life expectancy (7). Notably, aberrant RNLS expression has been documented in several malignancies, yet its specific expression patterns, biological functions, regulatory mechanisms, and potential interactions with key signaling pathways, particularly STAT3, within the context of OC remain inadequately explored, constituting a significant knowledge gap warranting thorough investigation.

Signal transducer and activator of transcription 3 (STAT3) is a key mediator that connects extracellular stimuli to transcriptional regulation and plays a pivotal role in maintaining OC stemness (8, 9). Colomiere et al. observed that aberrant STAT3 activation in OC correlates with peritoneal metastasis, wherein interactions with growth factor receptors (e.g., EGFR) or cytokine receptors (e.g., IL-6R) drive EMT-mediated tumor cell survival and invasion (10). The PI3K/AKT pathway, a classical oncogenic axis, its dysregulation is a hallmark of ovarian carcinoma (11). Emerging evidence indicates that RNLS functions as an oncogenic factor, promoting pancreatic cancer progression through the activation of the PI3K/AKT signaling axis (12). Emerging evidence indicates that this pathway suppresses ferroptosis by upregulating antioxidants such as glutathione (GSH) and glutathione peroxidase 4 (GPX4) (13). In addition, a study by Hasan et al. have demonstrated that STAT3 regulates its downstream signaling pathway, PI3K/AKT, to promote the growth of OC cells (14). In OC, the STAT3 pathway is also frequently constitutively active, with its activation level strongly correlating with tumor aggressiveness, chemoresistance, and poor clinical outcomes. However, the key upstream regulators driving persistent STAT3 activation in OC and their precise mechanisms of action require further in-depth elucidation.

In OC, chemotherapy-induced oxidative stress triggers ferroptosis, yet excessive reactive oxygen species (ROS) exacerbate ovarian dysfunction (15). Liu et al. demonstrated that PI3K/AKT alleviates oxidative stress by reducing intracellular malondialdehyde (MDA) and ROS levels, thereby attenuating ferroptosis (16). Furthermore, the roles of oxidative stress and ferroptosis in tumorigenesis, progression, and therapeutic resistance are gaining increasing recognition. Oxidative stress refers to a state of imbalance between intracellular ROS production and elimination, leading to excessive ROS accumulation that causes oxidative damage to lipids, proteins, and DNA. Ferroptosis is an iron-dependent, regulated form of cell death triggered by the accumulation of lipid peroxides, characterized by glutathione (GSH) depletion, loss of glutathione peroxidase 4 (GPX4) activity, and accumulation of the lipid peroxidation end-product MDA (17). Intriguingly, STAT3 inactivation may disrupt antioxidant defenses by dysregulating ferroptosis-associated genes like GPX4 (18), and functional crosstalk between PI3K/AKT and STAT3 activation has been documented in multiple cancers (19, 20). This multilayered regulatory network suggests that oncogenic pathways may reconfigure cell death thresholds via transcription factor networks, though the precise role of STAT3 in this context remains unclear.

This study aims to investigate RNLS-mediated regulation of STAT3 and its interplay with the PI3K/AKT pathway and ferroptosis-related molecular networks. Our findings may provide a rationale for developing combination therapies targeting this axis, including small-molecule inhibitors and ferroptosis inducers, to improve clinical outcomes in ovarian carcinoma patients.

## 2 Materials and methods

### 2.1 Cell culture and maintenance

Human OC cell lines (OVCAR3, SKOV3, ES2) from Procell (Wuhan, China), IGROV-1 from Cellverse (Shanghai, China), and normal ovarian epithelial cells (IOSE80) from Stemrecell (Shanghai, China) were cultured in RPMI 1640 (Gibco, Thermo Scientific, MA, USA) or McCoy’s 5A (Gibco) medium supplemented with 10% fetal bovine serum (FBS, Gibco) and 1% penicillin/streptomycin (Meilunbio, Dalian, China) at 37°C. Subculturing was performed using trypsin-EDTA (Meilunbio) upon reaching 80–90% confluency. Cells were passaged at a ratio of 1:3 and maintained in T25 flasks or multiwell plates as required for subsequent experiments. Media were refreshed every 48 hours to ensure optimal growth conditions.

### 2.2 Lentiviral transduction for RNLS knockdown and overexpression

Stable RNLS-knockdown (sh-RNLS) and overexpression (ov-RNLS) OVCAR3 cell models were generated using third-generation lentiviral vectors. For viral packaging, 293T cells (ATCC^®^ CRL-3216™) were co-transfected with transfer plasmids (pLKO.1-puro for shRNA or pLVX-IRES-ZsGreen1 for overexpression, Addgene, MA, USA) and packaging plasmids (psPAX2 and pMD2.G, Addgene) using Lipofectamine 3000 (Invitrogen, Thermo Scientific) at a ratio of 4:3:1 (plasmid: psPAX2: pMD2.G). Viral supernatants were harvested at 48 and 72 hours post-transfection, filtered through 0.45 μm PVDF membranes (Beyotime, Shanghai, China), and concentrated via ultracentrifugation (50,000 ×*g*, 4°C, 2 hours). Viral titers were determined by ELISA quantification of p24 antigen (1×10^8^ TU/mL, Takara, Dalian, China). For RNLS knockdown, three independent shRNA sequences (sh-RNLS-1: 5′-AGCTGTTGCTGTTACTTTA-3′; sh-RNLS-2: 5′-GGATGTGTCCTTGAATCAA-3′; sh-RNLS-3: 5′-GCTTCGTCTCCATTGATAA-3′) targeting distinct regions of the RNLS transcript were cloned into pLKO.1-puro, with scrambled shRNA (sh-NC) as the negative control. For overexpression, the full-length human RNLS cDNA (NM_001031709.3) was subcloned into pLVX-IRES-ZsGreen1 under the CMV promoter, with an empty vector (ov-NC) serving as the control. OVCAR3 cells at 20% confluency in 6-well plates were transduced with concentrated lentiviral particles at a multiplicity of infection (MOI) of 20 in RPMI 1640 containing 8 μg/mL Polybrene (Beyotime). After 6 hours of incubation at 37°C, the virus-containing medium was replaced with fresh complete medium. Transduction efficiency was validated by quantitative real-time PCR (qPCR) analysis of RNLS expression.

### 2.3 Cell viability assay (CCK-8)

Cell viability was assessed using the CCK-8 assay (Meilunbio). OVCAR3 cells (5×10^4^ cells/mL) were seeded into 96-well plates and incubated for 24 hours. After treatment with STAT3 siRNA on sh/ov RNLS stable OVCAR3 cells, 10% CCK-8 reagent was added to each well and incubated for 40 minutes at 37°C. Post-treatment, fresh medium was replenished and cells were cultured until designated timepoints (24/48/72 hours) prior to CCK-8 assay. Absorbance at 450 nm was measured using a microplate reader (Thermo Scientific, Multiskan 3K). Data were normalized to untreated controls, and viability rates were calculated from triplicate wells across three independent experiments.

### 2.4 Transwell invasion and wound healing assays

Matrigel matrix (Corning, NY, USA) was thawed overnight at 4°C, diluted 1:8 in pre-chilled serum-free medium, and 50 μL aliquots (~1 mg/mL final concentration) were polymerized in Transwell chambers at 37°C for 1 hour. For invasion assays, OVCAR3 cells (2.5×10^5^ cells/mL) in serum-free medium were seeded into the upper chambers of 24-well Transwell inserts (Corning). The lower chambers contained 20% FBS. After 24 hours, non-invading cells were removed with a cotton swab, and invaded cells were fixed with 4% paraformaldehyde, stained with 0.1% crystal violet (Meilunbio), and imaged under a microscope (Ts2-FL, Nikon, Japan). For wound healing assays, confluent monolayers in 6-well plates were scratched with a sterile pipette tip, washed with PBS, and cultured in 2% FBS medium. Five representative fields (×100 magnification) per wound were imaged at identical locations immediately post-scratching (0h) and at 24h. Migration distances were quantified at 0 and 24 hours using ImageJ software (National Institutes of Health [NIH], MD, USA).

### 2.5 Flow cytometry for apoptosis and ROS detection

Apoptosis was analyzed using the Annexin V-APC/PI Apoptosis Kit (AP107, Lianke, Hangzhou, China). Cells were harvested, washed with PBS, and resuspended in 1× binding buffer. After staining with Annexin V-APC and PI for 15 minutes in the dark, samples were analyzed on a BD FACSCalibur flow cytometer (BD Biosciences, CA, USA). For ROS detection, cells were incubated with 10 μM dihydroethidium (DHE, Beyotime) for 30 minutes, washed, and analyzed via flow cytometry at 488 nm excitation/610 nm emission. Data were processed using FlowJo software (version 12.0; FlowJo LLC), with gating based on unstained and single-stained controls.

### 2.6 Western blot analysis

Total proteins were extracted from cells or tissue using RIPA lysis buffer (Meilunbio). Protein concentrations were determined via BCA assay (Meilunbio). Equal amounts of protein (20 μg) were separated on 10% SDS-PAGE gels and transferred to PVDF membranes (Beyotime). Membranes were blocked with 5% non-fat milk (Beyotime) and probed overnight at 4°C with primary antibodies against RNLS (1:500, 15003-1-AP, Proteintech, Wuhan, China), STAT3 (1:2000, 10253-2-AP, Proteintech), p-STAT3 Tyr705 (1:1000, ab267373, Abcam, Cambridge, UK), p-STAT3 Ser727 (1:1000, ab32143, Abcam), PI3K (1:5000, 60225-1-Ig, Proteintech), p-PI3K (1:500, bs-6417R, Bioss, Beijing, China), AKT (1:5000, 60203-2-Ig, Proteintech), p-AKT (1:2000, 66444-1-Ig, Proteintech), and GAPDH (1:50000, 60004-1-Ig, Proteintech). Membranes were washed thrice in TBST (0.1% Tween-20, 10 min/wash), incubated with HRP-conjugated secondary antibodies (1:2000, SA00001-2, Proteintech), and developed using ECL kit (Meilunbio). Band intensities were quantified via ImageJ densitometry, with target protein expression normalized against GAPDH.

### 2.7 qPCR assay

Total RNA was extracted from cells or tissue with RNAiso Plus (Takara) and reverse-transcribed into cDNA using the NovoScript^®^ Plus cDNA Synthesis Kit (Novoprotein, Suzhou, China). qPCR was performed on an ABI 7300 system with NovoStart^®^ SYBR SuperMix (Novoprotein). The qPCR protocol included an initial denaturation at 95°C for 1 minute, followed by 40 cycles of 20 s at 95°C for denaturation, 20 s at 56°C for annealing, and 38 s at 72°C for extension. Primers for RNLS (Mice, F: 5′-AGAAGTCTCCCTCAAGCACTG-3′, R: 5′-GCATGGTGAGGATGACAAGGT-3′; Human, F: 5′-GCTCCCCTGAGCAGTTTGAT-3′, R: 5′-TTGCTGCCTTTGGCATTCAC-3′) and STAT3 (Mice, F: 5′-AGCTGGACACACGCTACCT-3′, R: 5′-AGGAATCGGCTATATTGCTGGT-3′; Human, F: 5′-AGAAGGACATCAGCGGTAAGA-3′, R: 5′-GGATAGAGATAGACCAGTGGAGAC-3′) were synthesized by Shangya Biotechnology (Zhejiang, China). GAPDH (Mice, F: 5′-GGTGAAGGTCGGTGTGAACG-3′, R: 5′-CTCGCTCCTGGAAGATGGTG-3′; Human, F: 5′-GCTCATTTGCAGGGGGGAG-3′, R: 5′-GTTGGTGGTGCAGGAGGCA-3′) served as the endogenous control. Relative expression was calculated using the 2^−ΔΔCt^ method (21).

### 2.8 Immunofluorescence assay

STAT3 subcellular localization was analyzed using IF. OVCAR3 cells grown on glass coverslips were fixed with 4% paraformaldehyde for 20 min, permeabilized with 0.5% Triton X-100 (Beyotime) for 15 min. Cells were incubated overnight at 4°C with STAT3 primary antibody (1:50), followed by Coralite594-conjugated goat anti-rabbit IgG (1:100, SA00013-4, Proteintech) for 1 h at 37°C. Nuclei were counterstained with DAPI (1 μg/mL, Meilunbio) for 5 min. Coverslips were mounted with anti-fade reagent (Meilunbio) and imaged using a Nikon Ts2-FL fluorescence microscope with excitation/emission filters set for 594 nm (red) and 358 nm (blue).

### 2.9 Transmission electron microscopy

Mitochondrial ultrastructure was examined by TEM. Cells were fixed in 2.5% glutaraldehyde overnight at 4°C, post-fixed with 1% osmium tetroxide for 2 h, and dehydrated through an ethanol gradient (50%-100%). Samples were embedded in Epon 812 resin and sectioned into 70-nm ultrathin slices using a ultramicrotome (Leica UC7, Leica, Germany). Sections were stained with uranyl acetate and lead citrate for 15 min each. Images were acquired using a Hitachi HT7800 TEM operated at 120 kV, with mitochondrial cristae structure and membrane integrity analyzed using ImageJ software.

### 2.10 *In vivo* xenograft tumor model

Male BALB/c nude mice (5-week-old) were procured from Sibeifu Biotechnology Co., Ltd. and housed under specific pathogen-free conditions with a 12-hour light/dark cycle. Mice were randomized into 4 groups (n=6 per group): sh-NC, sh-RNLS, ov-NC, and ov-RNLS. Subcutaneous tumors were induced by injecting 5.5 × 10^6^ OVCAR3 cells (suspended in 100 μL PBS) into the right flank. Tumor dimensions (length and width) were measured every 5 days using digital calipers, and volumes were calculated as V = 1/2×(length×width^2^), with timepoints recorded at days 5, 10, 15, 20, and 25 post-inoculation. At experimental endpoint (day 25), humane euthanasia was achieved through tail vein administration of sodium pentobarbital (200 mg/kg). Excised tumors underwent gravimetric analysis using a precision balance (BS210S, Sartorius) followed by bifurcated processing: 4% paraformaldehyde fixation (48h) for histopathological evaluation versus cryopreservation in liquid nitrogen for downstream molecular profiling. All experimental were approved by the Institutional Animal Care and Use Committee of Fujian Cancer Hospital (approval No. IACUC FJABR 2024080101).

### 2.11 Immunohistochemistry

Tumor tissues from xenograft models were fixed in 4% paraformaldehyde, paraffin-embedded, and sectioned at 4 μm. After deparaffinization and antigen retrieval with citrate buffer, sections were blocked with 5% BSA and incubated overnight with antibodies against Ki-67 (1:1000, 28074-1-AP, Proteintech), RNLS (1:50), or STAT3 (1:20). HRP-conjugated secondary antibodies and DAB substrate were applied for signal detection. Slides were counterstained with hematoxylin (Beyotime), dehydrated, and mounted with neutral resin. Images were captured using a microscope (E200, Nikon) and observe via Fully automatic digital slide scanner (version GCell-60, GCell, Guangzhou, China).

### 2.12 GSH, MDA, and GPX4 measurements

Reduced GSH and MDA levels in tumor tissues were quantified using commercial kits (Jiancheng Bioengineering Institute, Nanjing, China). Tissue samples (50 mg) were homogenized in ice-cold PBS (1:9 w/v), centrifuged at 12,000×g (4°C, 15 min), and supernatants collected. For GSH, homogenates were mixed with precipitating reagent and chromogenic substrates, and absorbance was measured at 405 nm. MDA was assessed via thiobarbituric acid reactive substances assay at 532 nm. GPX4 activity was determined using an ELISA kit (MM-60328H2, Enzyme Immunoassay). Protein concentrations were normalized using BCA assay results.

### 2.13 Statistical analysis

Data are presented as mean ± SD from three independent experiments. Comparisons between groups were analyzed using one-way ANOVA followed by Tukey’s *post hoc* test in GraphPad Prism 8.0. A p-value <0.05 was considered statistically significant.

## 3 Results

### 3.1 RNLS is upregulated in OC cells and modulates STAT3 expression

**Figure 1:**
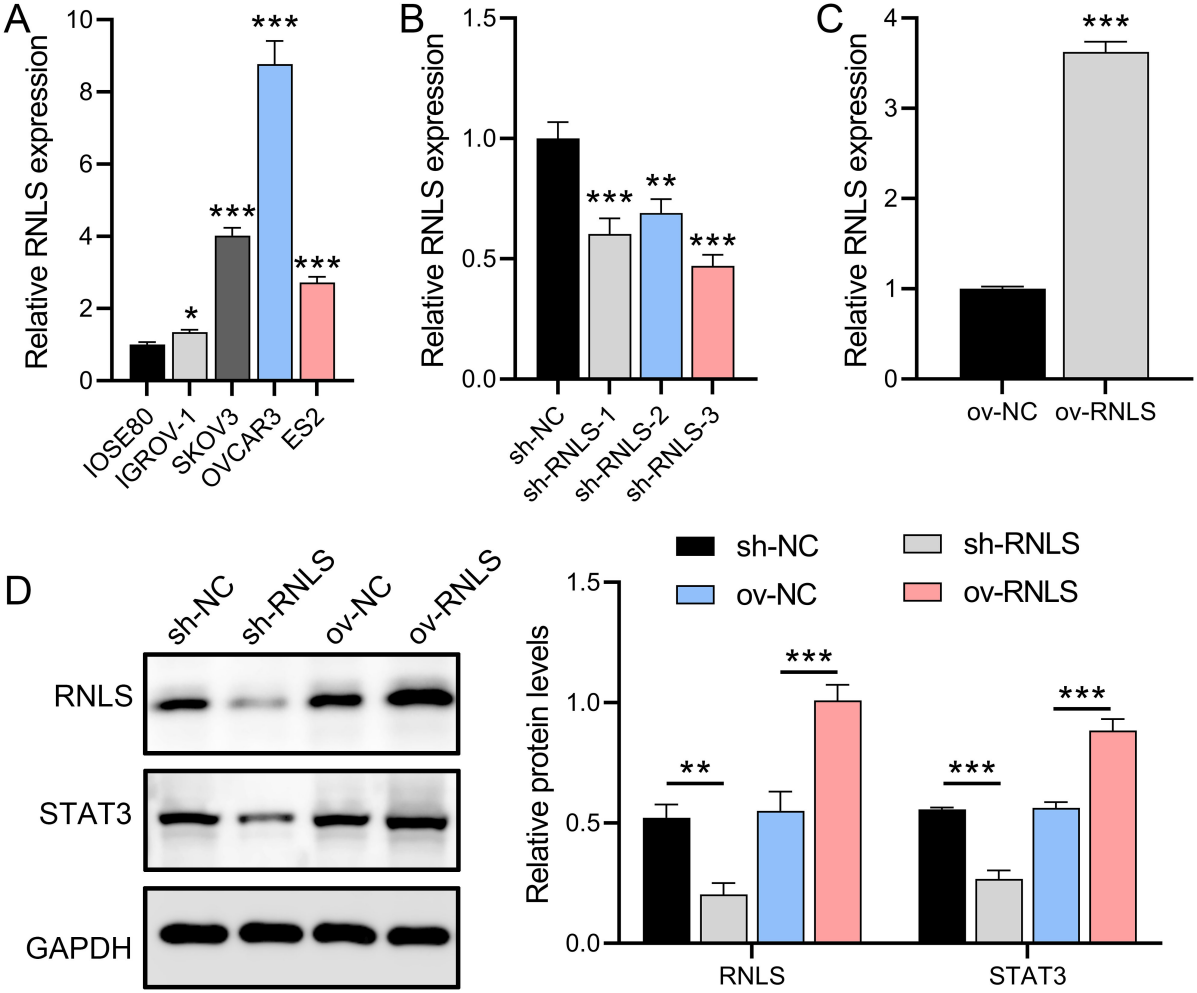
RNLS expression modulation and STAT3 association in OC cells. **(A)** qPCR analysis of RNLS mRNA levels in IOSE80 normal ovarian epithelial cells and OC cell lines. **(B, C)** Validation of RNLS knockdown (sh-RNLS) and overexpression (ov-RNLS) in OVCAR3 cells by qPCR. **(D)** Western blot analysis of RNLS and STAT3 protein levels in modulated cell lines. *P<0.05, **P<0.01, ***P<0.001.

OC cell lines relative to normal ovarian epithelial cells. Among these, the OVCAR3 line exhibited the most marked RNLS upregulation (**Figure 1A**). To interrogate RNLS functionality, stable OVCAR3 derivatives with either RNLS knockdown (sh-RNLS) or overexpression (ov-RNLS) were generated via lentiviral transduction. Subsequent analyses confirmed successful transcriptional and translational modulation of RNLS in these engineered lines (**Figures 1B, C**). Western blot analysis revealed that RNLS and STAT3 protein levels were suppressed by sh-RNLS and promoted by ov-RNLS (**Figure 1D**). These results indicate that the expression of RNLS affects the transcription and translation of STAT3, which is a key factor influencing the OC process.

### 3.2 RNLS drives malignant phenotypes in OC cells

**Figure 2:**
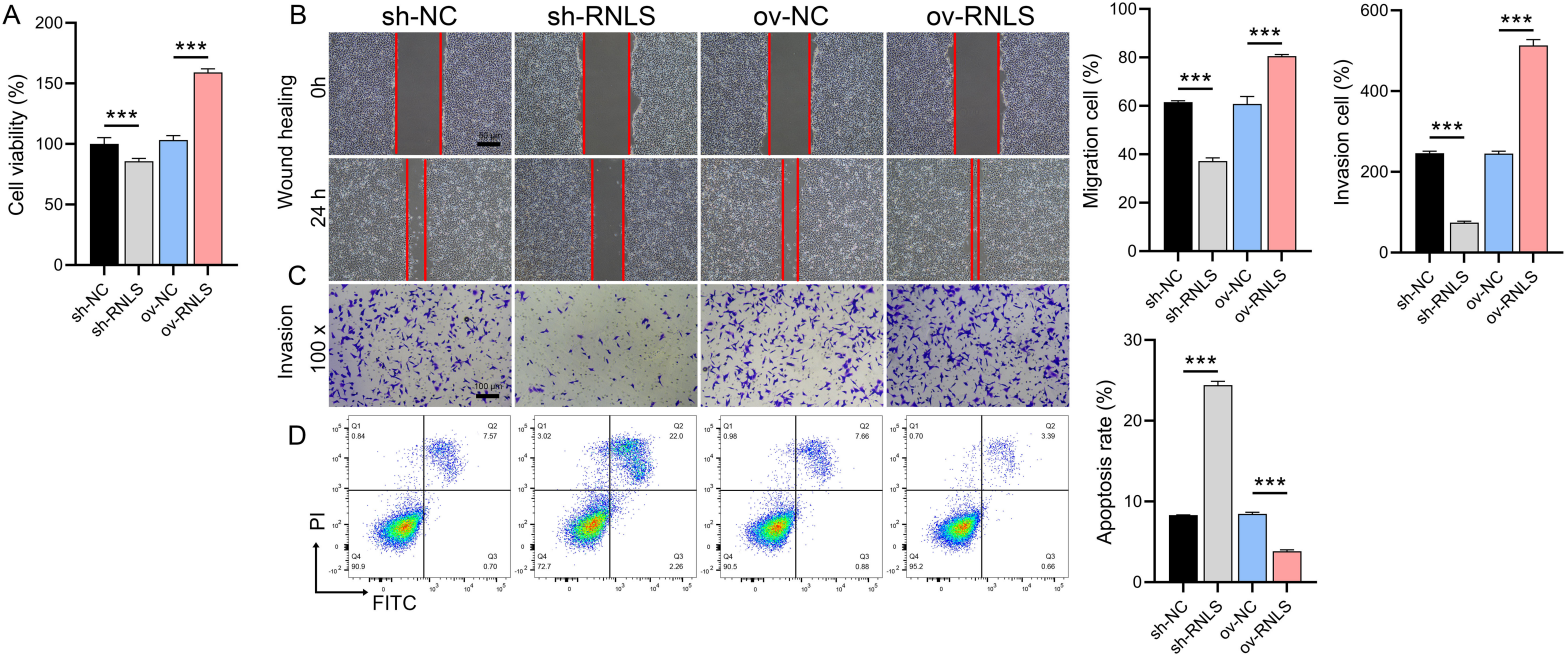
Functional effects of RNLS modulation on OC cells. **(A)** CCK-8 assay was performed to evaluate the effect of RNLS knockdown and overexpression on the viability of OVCAR3 cells. **(B)** Wound healing assay was conducted to assess the impact of RNLS modulation on the migratory capacity of OVCAR3 cells. **(C)** Transwell assay was used to determine the effect of RNLS knockdown and overexpression on the invasive potential of OVCAR3 cells. **(D)** Flow cytometry analysis was carried out to examine the influence of RNLS expression levels on apoptosis in OVCAR3 cells. ***P<0.001.

Functional assays demonstrated RNLS’s critical role in driving malignant behaviors. RNLS depletion significantly impaired cell viability in CCK-8 assays (**Figure 2A**), reduced wound closure capacity in migration assays (**Figure 2B**), and attenuated cellular invasion through Matrigel in Transwell assays (**Figure 2C**). Conversely, RNLS overexpression enhanced proliferation, accelerated migration, and increased invasion. Flow cytometry analysis further revealed that RNLS knockdown markedly elevated apoptosis rates, while RNLS overexpression conferred significant protection against apoptosis (**Figure 2D**). These results establish RNLS as a key regulator of OC cell proliferation, motility, invasion, and survival.

### 3.3 RNLS acts upstream of STAT3 to regulate PI3K/AKT signaling

**Figure 3:**
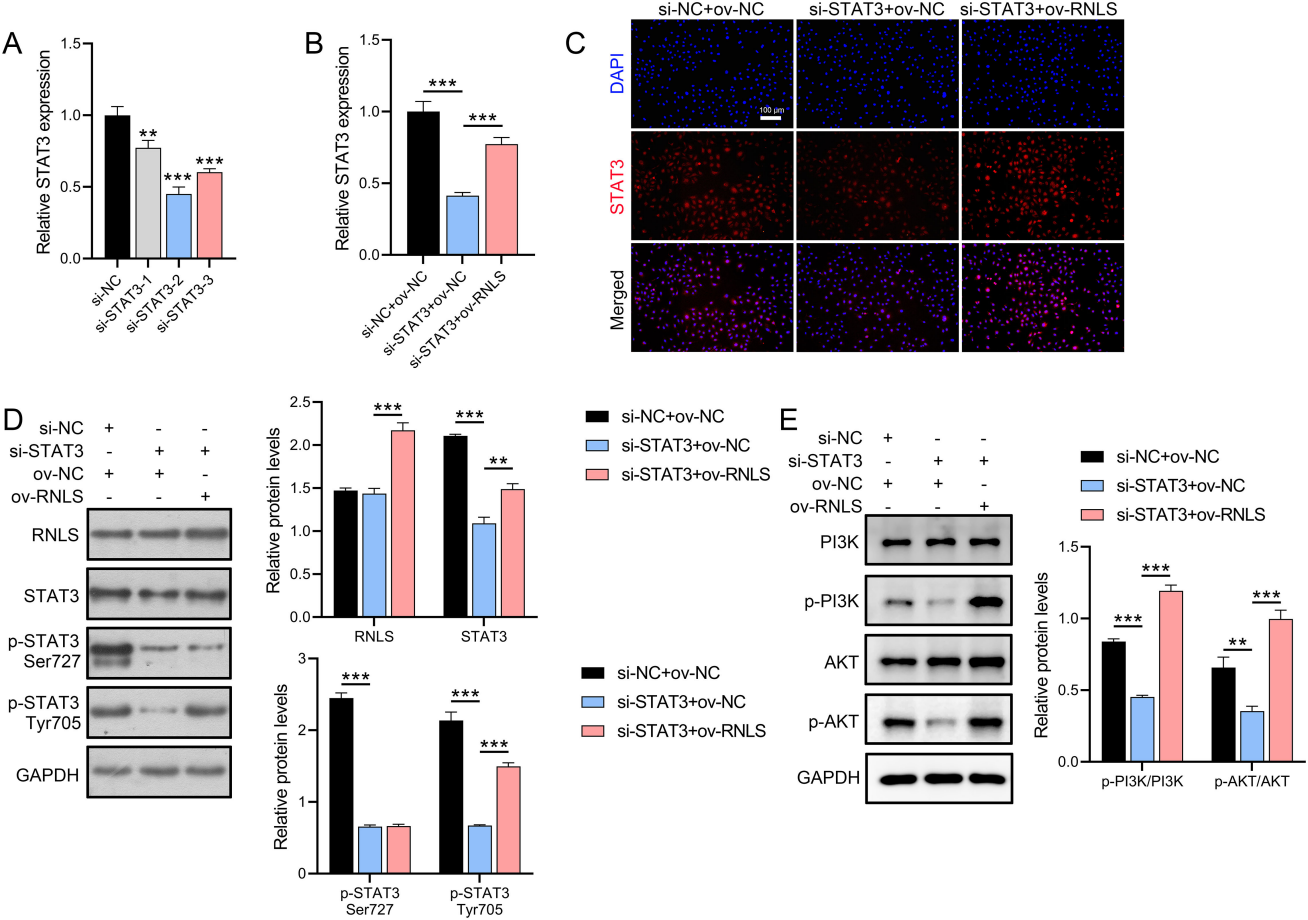
STAT3 silencing and RNLS overexpression interplay. **(A)** The inhibition efficiency of three si-STAT3 assays was verified by qPCR in OVCAR3 cells. **(B)** qPCR analysis of STAT3 expression in si-STAT3, ov-RNLS, and combination groups. **(C)** IF analysis of STAT3 nuclear localization in si-STAT3, ov-RNLS, and combination groups. **(D)** Western blot analysis of RNLS, STAT3, p-STAT3 Ser727 and p-STAT3 Tyr705. **(E)** Western blot analysis of p-PI3K, PI3K, p-AKT, and AKT protein levels in si-STAT3, ov-RNLS, and combination groups. **P<0.01, ***P<0.001.

To dissect the functional interplay between RNLS and STAT3, STAT3 expression was silenced in OVCAR3 cells using siRNA. Three STAT3-targeting siRNAs were transfected into OVCAR3 cells, with si-STAT3–2 showing the strongest suppression of STAT3 mRNA (**Figure 3A**). qPCR and IF analyses demonstrated that ov-RNLS rescued STAT3 expression suppressed by si-STAT3 (**Figures 3B, C**). Furthermore, Western blot analysis revealed that si-STAT3 transfection did not significantly affect RNLS protein levels but suppressed the expression of STAT3, p-STAT3 (Ser727), and p-STAT3 (Tyr705). In contrast, co-transfection with ov-RNLS promoted the protein levels of RNLS, STAT3, and p-STAT3 (Tyr705), while exhibiting no significant effect on p-STAT3 (Ser727) expression (**Figure 3D**). Furthermore, si-STAT3 inhibited p-PI3K/PI3K and p-AKT/AKT ratios, while ov-RNLS co-transfection mitigated this inhibition (**Figure 3E**). These results indicate that RNLS serves as an upstream regulator of STAT3 and selectively modulates its phosphorylation at the Tyr705 residue.

### 3.4 RNLS overexpression rescues malignant phenotypes in STAT3-depleted cells

**Figure 4:**
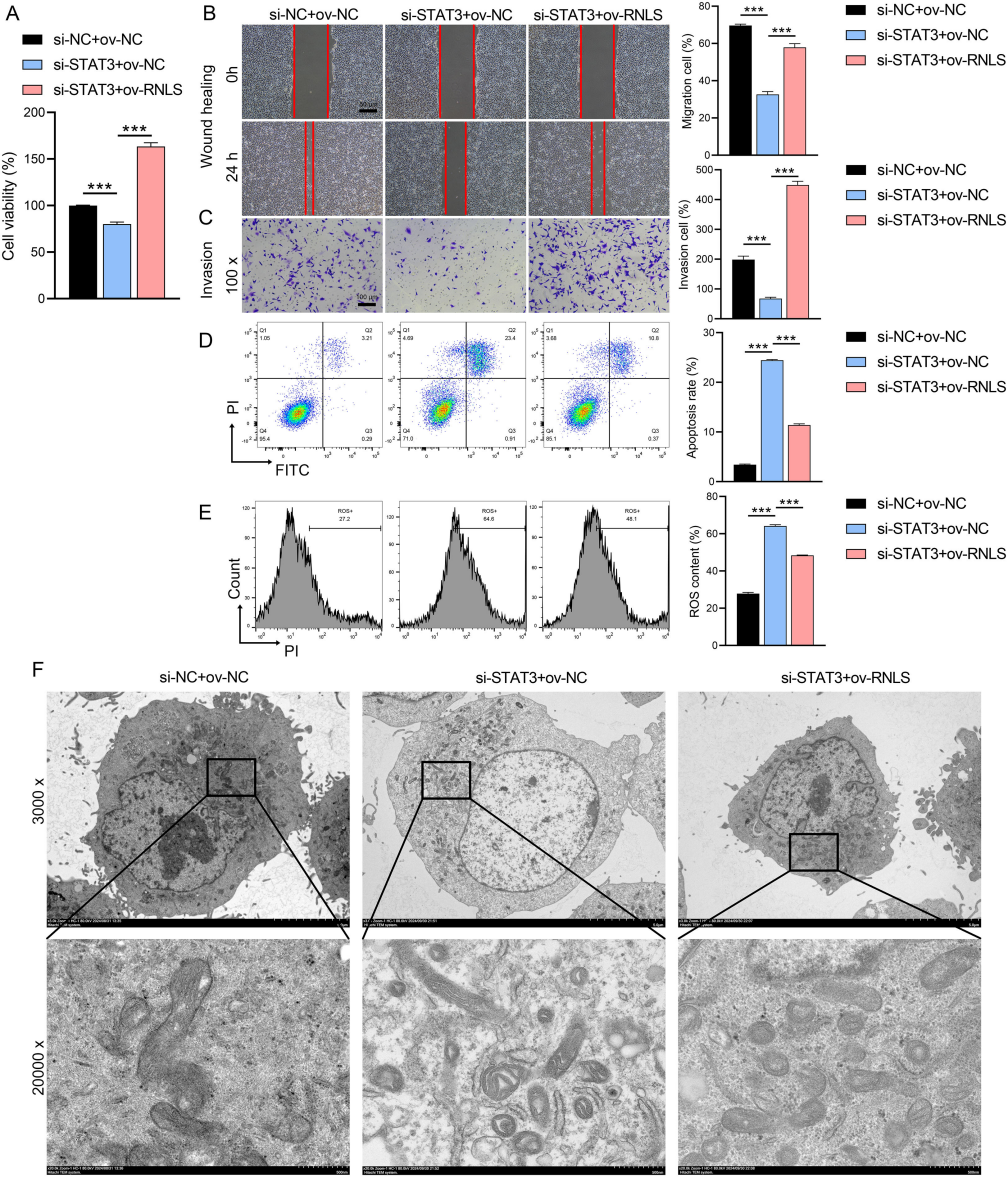
Rescue effects of RNLS overexpression on STAT3 silencing. **(A)** CCK-8 assay for viability in si-STAT3, ov-RNLS, and combination groups. **(B)** Wound healing assessing migration in si-STAT3, ov-RNLS, and combination groups. **(C)** Transwell assays assessing invasion recovery in si-STAT3, ov-RNLS, and combination groups. **(D)** Flow cytometry analysis of apoptosis in si-STAT3, ov-RNLS, and combination groups. **(E)** Flow cytometry analysis of ROS levels in si-STAT3, ov-RNLS, and combination groups. **(F)** TEM of mitochondrial ultrastructure in si-STAT3, ov-RNLS, and combination groups. ***P<0.001.

We next investigated whether RNLS could functionally compensate for STAT3 loss. STAT3 knockdown significantly impaired cell viability (**Figure 4A**), reduced migration and invasion capacities (**Figures 4B, C**), and increased apoptosis and intracellular reactive oxygen species (ROS) levels (**Figures 4D, E**). Strikingly, co-expression of ov-RNLS substantially mitigated these detrimental effects, partially restoring proliferation, migration, and invasion, while concurrently reducing apoptosis and ROS accumulation. TEM revealed that STAT3 deficiency induced severe mitochondrial damage, including cristae disorganization and vacuolization. RNLS co-expression significantly ameliorated these ultrastructural mitochondrial abnormalities (**Figure 4F**), indicating RNLS protects mitochondrial integrity downstream of STAT3.

### 3.5 The influence of Fer-1 on RNLS-STAT3 axis in oxidative stress

**Figure 5:**
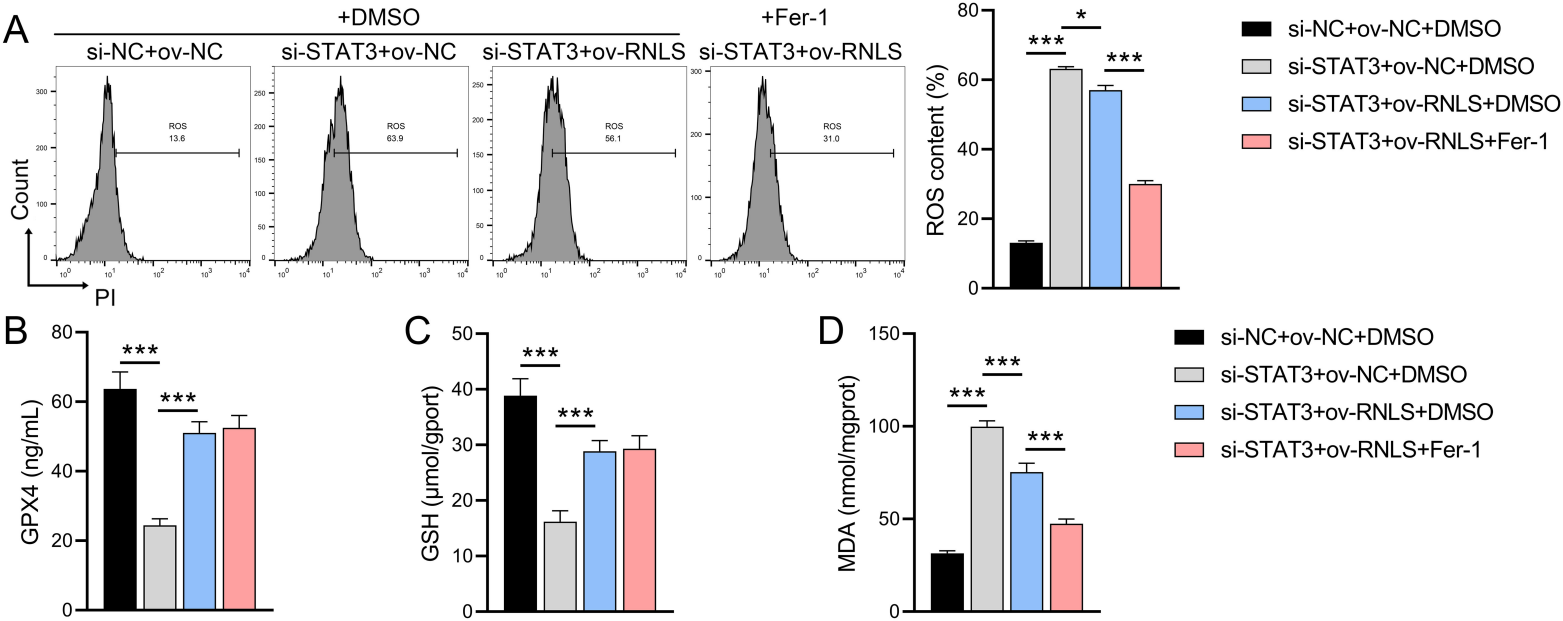
The influence of ferroptosis inhibitor Fer-1 on the role of the RNLS-STAT3 axis in oxidative stress. **(A)** Flow cytometry analysis of ROS levels in OVCAR3 cells following modulation of RNLS and STAT3 expression. **(B-D)** ELISA quantification of MDA, GPX4, and GSH levels in OVCAR3 cells co-transfected with sh-RNLS or ov-RNLS and si-STAT3. *P<0.05, ***P<0.001.

Given the observed ROS dysregulation, we probed the role of the RNLS-STAT3 axis in redox homeostasis and ferroptosis. We observed that STAT3 suppression significantly increased ROS levels, reduced GPX4 and GSH levels, and promoted MDA accumulation compared to controls (**Figures 5A–D**). These effects were reversed by ov-RNLS co-expression. Treatment with the ferroptosis inhibitor ferrostatin-1 (Fer-1) attenuated STAT3 knockdown-induced ROS and MDA accumulation but failed to restore GPX4 or GSH levels, suggesting RNLS regulates ferroptosis susceptibility through mechanisms distinct from or broader than Fer-1’s action, potentially involving direct GPX4/GSH modulation via the STAT3 pathway.

### 3.6 RNLS drives tumor growth and modulates redox markers *in vivo*

**Figure 6:**
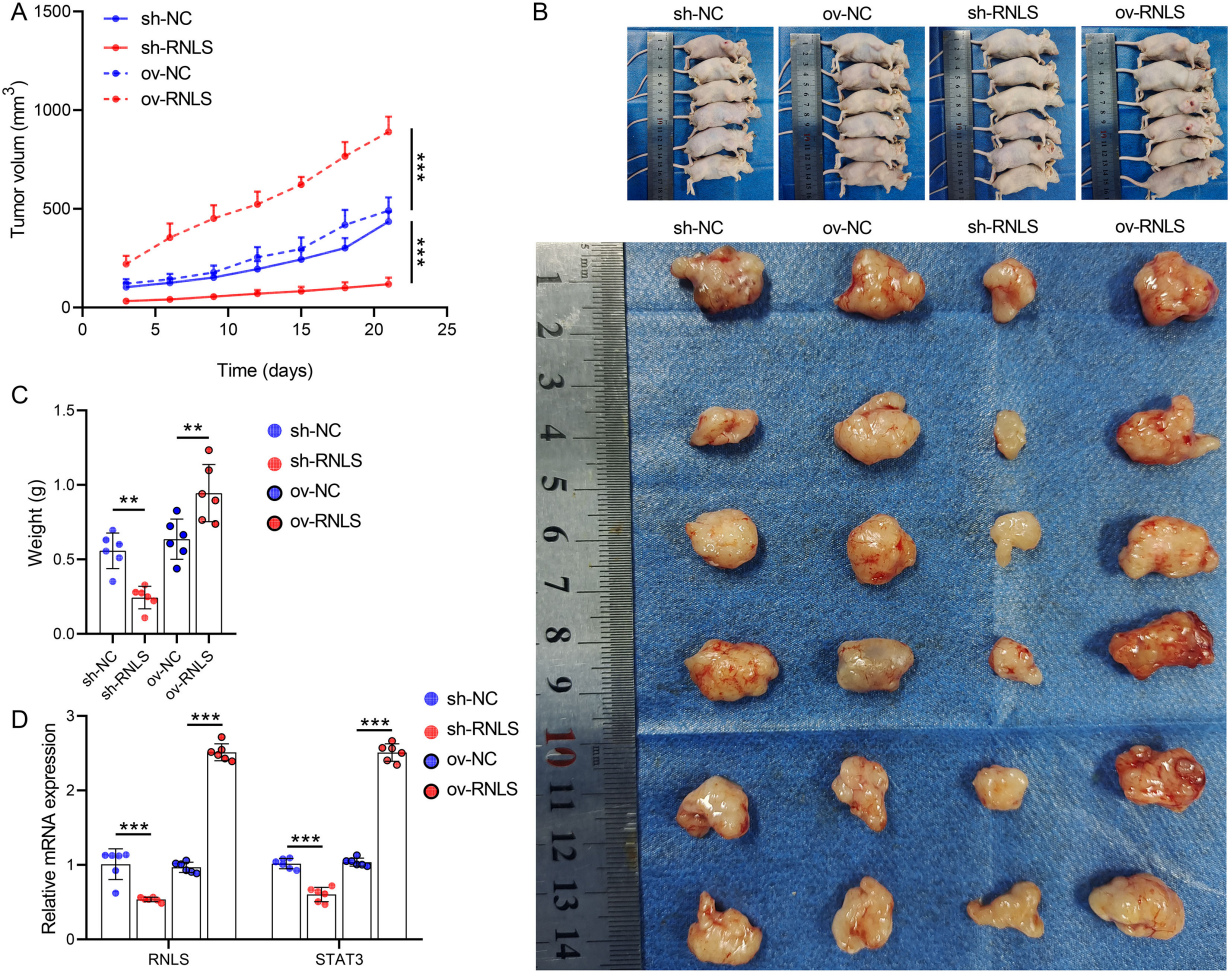
*In vivo* tumorigenic role of RNLS. **(A)** Subcutaneous tumor growth curves of OVCAR3 cells with RNLS knockdown or overexpression in a xenograft mouse model. **(B)** Representative tumor images. **(C)** Tumor weights post-excision. **(D)** qPCR validation of RNLS and STAT3 expression levels in tumor tissues with RNLS knockdown or overexpression. **P<0.01, ***P<0.001.

**Figure 7:**
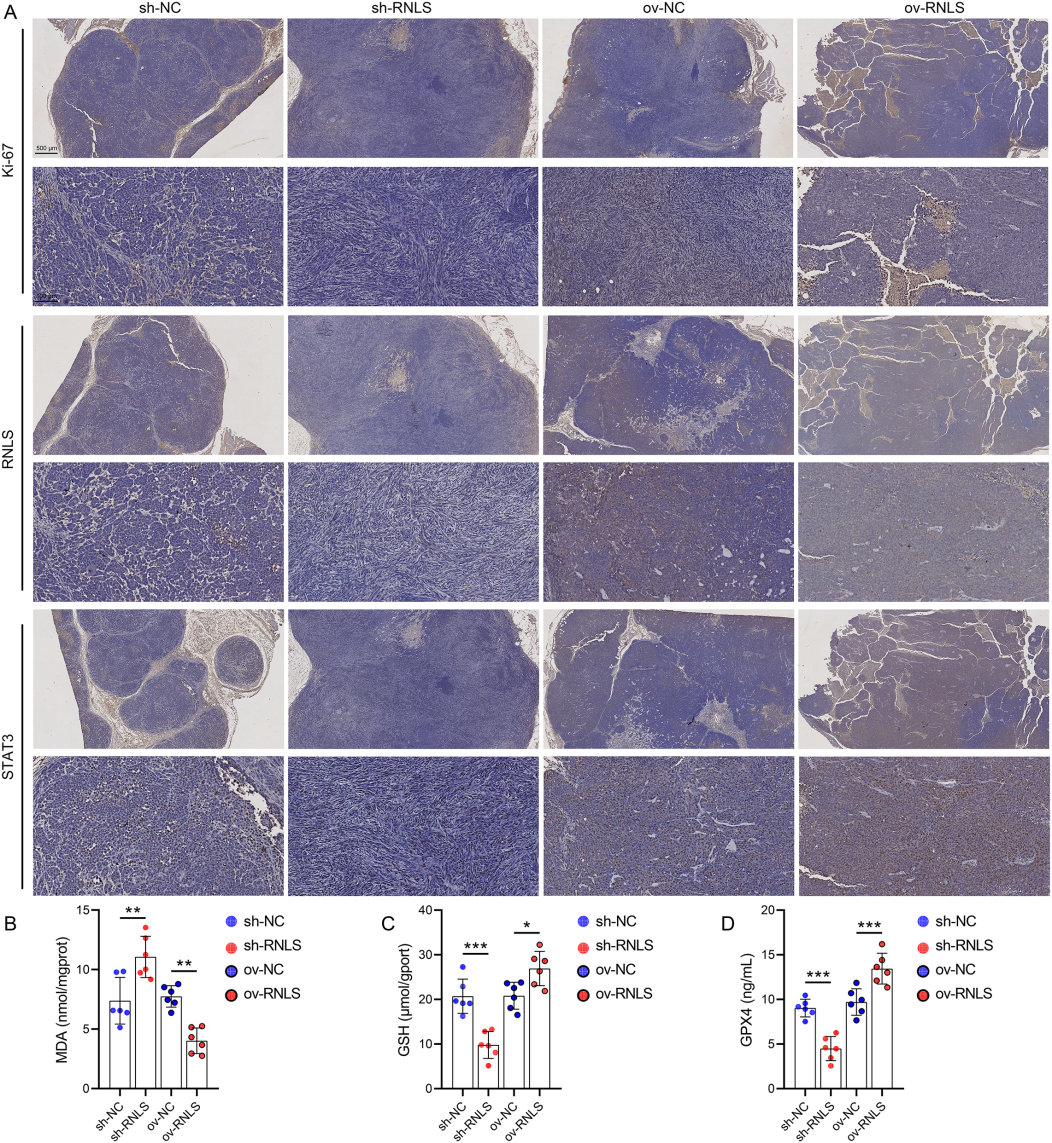
Biomarker profiling in xenograft tumors. **(A)** IHC staining of Ki-67, RNLS, and STAT3 in tumor tissues with RNLS knockdown or overexpression. **(B-D)** ELISA quantification of MDA, GPX4, and GSH levels in tissue homogenates from RNLS-knockdown and RNLS-overexpressing tumors. *P<0.05, **P<0.01, ***P<0.001.

Subsequently, we verified the pathophysiological significance of RNLS in the subcutaneous xenograft model. Tumors derived from sh-RNLS OVCAR3 cells exhibited significantly slower growth kinetics (**Figure 6A**) and lower final weights (**Figures 6B, C**) compared to control tumors. Conversely, tumors from ov-RNLS cells grew faster and were heavier. Molecular analysis of excised tumors confirmed downregulation of RNLS and STAT3 mRNA in sh-RNLS tumors and upregulation in ov-RNLS tumors (**Figure 6D**). IHC corroborated these findings, showing reduced protein expression of RNLS, STAT3, and the proliferation marker Ki-67 in sh-RNLS tumors, with opposite trends in ov-RNLS tumors (**Figure 7A**). Biochemical assays further revealed elevated MDA content and suppressed GPX4 activity/GSH levels in sh-RNLS tumors, mirroring the *in vitro* oxidative stress phenotype, while ov-RNLS tumors showed reduced MDA and enhanced GPX4/GSH activity (**Figures 7B–D**). These *in vivo* data conclusively demonstrate RNLS’s oncogenic role through STAT3-dependent modulation of tumor growth and redox balance.

## 4 Discussion

As the most lethal disease among gynecological malignancies, the molecular mechanism of OC, which is highly heterogeneous and treatment-resistant, has always been the focus of tumor biology research (22). In this study, we found that RNLS showed significantly high expression in OC cell lines, especially in OVCAR3 cells, a phenomenon suggesting that RNLS may be involved in the disease process as an oncogene. It has been well documented that STAT3 is one of the key pathways promoting OC progression and drug resistance (23, 24). By constructing cell models with stable knockdown and overexpression of RNLS, we observed a bidirectional regulation of STAT3 protein expression by RNLS: knockdown of RNLS significantly suppressed the protein level of STAT3, while overexpression enhanced its expression. This finding demonstrates for the first time the existence of a direct regulatory relationship between RNLS and STAT3 in OC and provides a new perspective for understanding the aberrant activation of the JAK-STAT pathway. Notably, as a key hub linking extracellular signaling with intranuclear transcriptional regulation, the activity status of STAT3 directly affects the proliferation, survival, and metabolic reprogramming of tumor cells (25). In this study, the regulatory effect of RNLS on STAT3 was not only reflected at the protein expression level, but also confirmed by IF experiments to promote the nuclear translocation process of STAT3. This spatially localized regulation may enhance the transcriptional activity of STAT3 by increasing its binding efficiency to the promoter region of target genes.

This study revealed the cascading activation effect of the RNLS-STAT3 axis on the PI3K/AKT pathway: STAT3 silencing significantly reduced the phosphorylation levels of PI3K and AKT, and RNLS overexpression was able to reverse this inhibitory effect, suggesting that STAT3 plays a role as a signaling node downstream of RNLS. As a classical pro-survival signaling pathway, PI3K/AKT pathway activation can promote the malignant phenotype of tumors through a multidimensional mechanism: on the one hand, it inhibits the expression of pro-apoptotic proteins by phosphorylating FOXO transcription factors (26), and on the other hand, it enhances protein synthesis and energy metabolism by activating the mTORC1 complex (27). The novelty of this study lies in the identification of a regulatory mode of the PI3K/AKT pathway mediated by the RNLS-STAT3 axis. si-STAT3-induced suppression of PI3K/AKT signaling was at least partially restored by RNLS overexpression. This restoration is attributed to the dual activation of STAT3 expression and its phosphorylation at the Tyr705 residue by RNLS, which drives downstream oncogenic PI3K/AKT signaling. This observation holds significant biological implications. Phosphorylation at Tyr705 is a canonical driving force and a key hallmark of STAT3 dimerization, nuclear translocation, and transcriptional activation, directly linked to its function in transcriptional regulation (28, 29). In contrast, phosphorylation at Ser727 is generally considered to fine-tune STAT3’s transcriptional activity or to be involved in non-transcriptional functions. Therefore, the specific enhancement of Tyr705 phosphorylation by RNLS strongly suggests that RNLS primarily exerts its functional effects through augmenting the classical transcriptional activity of STAT3. Elucidation of this regulatory mechanism provides a novel theoretical framework for understanding the frequent activation of the PI3K/AKT pathway in OC.

Ferroptosis as a programmed death mode, which has attracted much attention in recent years, has gradually become a research hotspot for its interaction with oncogenic signaling pathways. In this study, we found that the RNLS-STAT3 axis promotes the biosynthesis of GPX4 and GSH by activating the PI3K/AKT pathway, which contributes to the enhancement of cellular resistance to ferroptosis and antioxidant defense (30). In addition, this signaling reduces the accumulation of lipid peroxides MDA by maintaining mitochondrial structural integrity. We observed by electron microscopy that STAT3 knockdown led to structural disintegration and vacuolization of mitochondrial cristae, whereas RNLS overexpression significantly alleviated this damage, suggesting that STAT3 may maintain mitochondrial membrane stability by regulating the expression or modification of mitochondrial membrane proteins (e.g., VDAC or ANT family members) (31). In addition, ROS level assays indicated that STAT3 knockdown resulted in an intracellular ROS burst, an oxidative stress state that, together with decreased GPX4 activity and upregulated ACSL4 expression, constitutes a triggering condition for ferroptosis. Notably, the regulation of ferroptosis by the PI3K/AKT pathway may be tissue-specific, and the STAT3-dependent regulatory pattern identified in this study complements the mechanism of direct phosphorylation of GPX4 by AKT reported in hepatocellular carcinoma, highlighting the complexity and context-dependence of the pathway network in the regulation of cell death. Our rescue experiments demonstrate that PI3K/AKT activation is both necessary and sufficient to suppress ferroptosis triggered by STAT3 inhibition. This is evidenced by the reversal of ROS accumulation, mitochondrial damage, and GPX4/GSH downregulation—while apoptosis remained STAT3-dependent but PI3K/AKT-independent. Thus, PI3K/AKT specifically governs ferroptosis resistance downstream of RNLS-STAT3.

*In vivo* experiments, RNLS knockdown significantly inhibited the growth of subcutaneous transplanted tumors, which was accompanied by downregulation of Ki-67 expression as well as elevated MDA levels, which was highly consistent with the proliferation inhibition and ferroptosis activation phenotypes observed in the *in vitro* experiments. The intensity of nuclear staining of Ki-67, a core marker of cell proliferation, directly reflected the proliferative activity of tumor cells, and its high expression in OC was associated with tumor grade, metastatic potential and poor prognosis (32, 33). And the increase in tumor volume and weight in the RNLS overexpression group was correlated with the upregulation of GPX4 and GSH levels, further validating the hypothesis that RNLS drives tumor progression through pro-proliferation and anti-ferroptosis. Interestingly, the changes in STAT3 expression in tumor tissues were identical to those of RNLS, and this consistency from *in vitro* to *in vivo* not only reinforces the pathological relevance of the RNLS-STAT3 axis, but also implies that STAT3 may act as an upstream mediator of Ki-67 transcriptional regulation, mediating the pro-carcinogenic effects of RNLS through the activation of proliferation-associated target genes (e. c-Myc or E2F1) (34, 35).

The present study has several limitations. First, the *in vivo* experiments used only a subcutaneously grafted tumor model, which could not simulate the effect of the *in situ* OC microenvironment on the function of the RNLS-STAT3 axis. Second, the screening of downstream effectors of the PI3K/AKT pathway has not been performed comprehensively, especially the regulation of the mTORC1/4E-BP1 axis and glycolysis-related enzymes remain to be verified. Third, the specific mechanisms linking the non-transcriptional functions of STAT3 (e.g., mitochondrial localization-related roles) to ferroptosis resistance have not been elucidated; fourth, the correlation between RNLS expression and patient prognosis and chemotherapy resistance in clinical samples lacks data support. Finally, the development of small molecule inhibitors or gene editing tools targeting the RNLS-STAT3 axis has not been carried out, limiting the progress of translational research.

## 5 Conclusion

In conclusion, the present study systematically elucidated the molecular mechanism by which RNLS promotes OC progression through activation of the STAT3-dependent PI3K/AKT signaling pathway, and revealed the dual mode of action of this regulatory axis to inhibit ferroptosis by enhancing antioxidant defenses and preserving mitochondrial function. These findings not only enrich the theoretical system of the OC signaling network, but more importantly, provide an experimental basis for the development of targeted therapeutic strategies against the RNLS-STAT3 axis, thus opening new therapeutic avenues to improve the prognosis of OC patients.

## Data Availability

The original contributions presented in the study are publicly available. This data can be found here: https://www.jianguoyun.com/p/DdNJWFAQ7dK8Cxj4vfUFIAA.
