# Correction: RNLS promotes ovarian cancer growth and inhibits ferroptosis via mediating STAT3

**DOI:** 10.3389/fonc.2025.1697794

**Published:** 2025-10-13

**Authors:** Liang Lin, Zuolian Xie, Peilin Zhong, Jian Chen, Ning Ma, Ling Li, An Lin, Li Chen

**Affiliations:** Department of Gynecology, Clinical Oncology School of Fujian Medical University, Fujian Cancer Hospital, Fuzhou, China

**Keywords:** ovarian cancer, RNLS, STAT3, PI3K/AKT, oxidative stress

The order of authors in the author list of the published paper was erroneously given as:

“Liang Lin†, Zuolian Xie†, Peilin Zhong, Jian Chen, Ning Ma, Ling Li, Li Chen* and An Lin*”

The correct author list reads:

“Liang Lin^†^, Zuolian Xie^†^, Peilin Zhong, Jian Chen, Ning Ma, Ling Li, An Lin*, Li Chen*”

The original version of this article has been updated.

